# Testing a Susceptible Population Density Among Other Explanatory Factors of African Swine Fever Spread in Wild Boar Using the Russian Federation Data, 2007–2023

**DOI:** 10.1155/tbed/6569042

**Published:** 2025-08-28

**Authors:** O. I. Zakharova, E. A. Liskova, N. A. Gladkova, I. V. Razheva, I. V. Iashin, A. A. Blokhin, D. V. Kolbasov, F. I. Korennoy

**Affiliations:** ^1^Federal Research Center for Virology and Microbiology - Branch in Nizhny Novgorod, Nizhny Novgorod, Russia; ^2^Federal Research Center for Virology and Microbiology, Volginsky, Russia; ^3^Federal Center for Animal Health (FGBI ARRIAH), Vladimir, Russia

**Keywords:** African swine fever, CART, population density, regression analysis, wild boar

## Abstract

This study aims to identify the role of various natural, socioeconomic, and demographic factors in the development of the African swine fever (ASF) epizootic among wild boar in the Russian Federation (RF) from 2007 to 2023. In this study, particular emphasis was placed on testing the significance of wild boar population density as a key factor contributing to the spread of ASF within this population. During the study period, 1711 outbreaks in wild boars were reported in the RF, accounting for 41.7% of all ASF outbreaks in the country. We tested two regression approaches to model the dependance of the total number of ASF outbreaks in second-level municipal units (districts) on a range of potential explanatory factors, including the dynamically changing annual population density of wild boar. We employed negative binomial regression (NBR) and, as an alternative approach, classification and regression trees (CARTs). The predictive capabilities of both models were evaluated using 10-fold cross-validation. One of the most significant identified factors was the number of ASF outbreaks in domestic populations, which may indicate a close coexistence of both domestic and wild ASF cycles. Population density showed limited significance in the negative binomial model (*p*=0.05). The CART model demonstrated high significance for this factor in the Far Eastern regions of the country, where the highest number of outbreaks occurred at density values above 0.120 individuals/km^2^. For the European part of the RF, the threshold density value was 0.026 individuals/km^2^, which closely corresponds to the threshold established by country's authorities for managing wild boar populations to prevent the spread of ASF. The results demonstrated a complex and nonlinear influence of wild boar population density and ASF outbreaks among domestic pigs on the likelihood of new infection foci emerging in the wild fauna. The modeling results indicated that although both types of models had comparable predictive capabilities, the CART approach provided better visualization and understanding of the analysis results. These findings can be used to optimize population management activities to regulate wild boar numbers in infection hotspots across different geographical areas delineated by the risk level of infection spread.

## 1. Introduction

African swine fever (ASF) is a transboundary viral disease that affects both domestic pigs and wild boar and causes significant damage to the pig farming industry in many countries. ASF can manifest in both large-scale and localized epidemics, and it is associated with various risk factors that must be considered when selecting appropriate surveillance and control strategies [1–3].

Current scientific discussions are actively addressing the role played by domestic pigs and wild boars in the emergence and spread of ASF in areas that have not yet been reported. Research and experiments aim to identify the contribution of each of these animal species to the chains of virus transmission, as well as to assess the potential and conditions for controlling and preventing the spread of infection [4, 5].

The spread of ASF occurs not only through direct contact between susceptible animals but also indirectly through contact with infected carcasses of wild boars or contaminated objects in the surrounding environment [6, 7]. The spread of ASF can be enabled by the movement of contaminated items, such as mammals or birds scavenging carcasses, or biting flies/ticks [8].

Understanding the various mechanisms of ASF transmission in terms of the wild boar–domestic pig-environment ecosystem will help specialists develop effective disease control strategies and minimize the risk of emerging new epidemics [9, 10].

Despite the efforts made by many countries to develop an effective vaccine against ASF, the current strategy for combating the disease is based on assessing the risk factors for the spread of the infection, strictly adhering to biosecurity measures in the management of domestic pigs and wild boar and applying culling and cleansing/disinfection in response to a confirmed outbreak. Biosecurity includes controlling the movement of animals, maintaining hygiene standards, disinfecting equipment and care items, as well as zoning areas. Additionally, an effective fight against ASF involves informational campaigns aimed at pig farmers, agricultural workers, hunters, and the general public regarding preventive measures and control of infection spread [11–13].

Risk factors contributing to the spread of the ASF virus among wild boar encompass a wide and diverse range of predictors that have not yet been thoroughly studied and are represented in various scenarios of epizootic development. These factors may include the dynamics of wild boar population trends, changes in habitat conditions, contact with other animal species, and the impact of human economic activities on the spread of the infection. A comprehensive study of these factors will enhance our understanding of ASF transmission mechanisms and help us develop optimal strategies for controlling the virus among wild boar, which is crucial for effective management of the disease [14–16].

Studying the theoretical foundations for determining threshold values of susceptible animals in the emergence of ASF is crucial for planning measures to reduce wild boar populations around infection foci and subsequent campaigns for population management in disease-free areas to prevent the spread of the infection [17].

The aim of this study was to assess the significance of wild boar population density and to evaluate other risk factors for the spread of ASF among wild boar in regions of the Russian Federation (RF) using regression analysis methods. In our work, we analyzed a wide range of independent variables, including environmental factors such as forest cover percentage, the density of major roads, and the percentage of water bodies, as well as demographic characteristics expressed through wild boar population density, human population density, density of settlements, and the number of hunted wild boars, carcasses, and remains of infected animals found, which have potential significance in the ASF epizootic in the RF. Of particular interest in our study was the factor of wild boar population density, as the confirmation of ASF in wildlife necessitates wild boar depopulation measures, which are implemented in infection hotspots to regulate animal numbers to the threshold value recommended by government authorities of 0.025 individuals/km^2^.

## 2. Materials and Methods

### 2.1. Study Area

For the study, model subjects (first-level administrative divisions) of the RF were selected based on the following criteria: (a) ASF outbreaks among wild boars were regularly registered during the analyzed period in the same districts; (b) the presence and availability of annual data on wild boar population numbers at the district level (second-level administrative divisions). The model region presented for regression analysis consisted of 39 subjects of the RF and was divided into two territories based on geographic criteria: the European territory, comprising 35 subjects, and the Far Eastern territory, including four subjects. The model subjects included 2440 districts, which were the units of analysis in our study.

### 2.2. ASF Data

**Figure 1:**
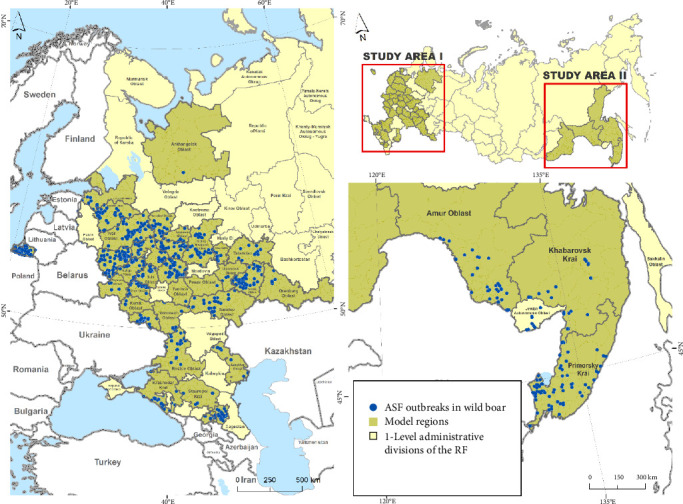
Epidemic situation of African swine fever in the model regions of Russia, 2007–2023.

Data on ASF outbreaks in the RF were obtained from the World Organization for Animal Health's animal disease notification database (WOAH WAHIS) for the period from 2007 (a year when the disease was first introduced to Russia) to 2023. During the study period, a total of 1711 ASF outbreaks in wild boars were registered in the model districts, with 1308 outbreaks noted in the European zone and 403 outbreaks in the Far Eastern region. In this study, we considered an “outbreak” as a case of ASF in wild boars confirmed by laboratory methods and notified to WOAH, defined by geographical coordinates and the date of occurrence [18] (Figure 1).

### 2.3. Explanatory Factors

The most significant factors that play a role in the spread of ASF among wild boar were selected as independent variables based on a literature review [18–22].

Data on wild boar population numbers in the studied districts from 2007 to 2023, as well as information on carcasses of animals that died from various reasons and their remains found during routine monitoring activity, were obtained from statistical reports of the regional ministries of natural resources and ecology of the RF (https://www.mnr.gov.ru/about/).

Environmental variables were gathered from the vector and raster GIS layers of Open Street Maps (OSMs) (https://www.openstreetmap.org/#map=3/69.62/-74.90). The landscape variables included the percentage of water bodies (rivers and lakes), the area and share of vegetation cover, and the length and density of roads. All variables were extracted and summed by district, and median values were calculated using GIS zonal statistics tools. Data on population density and the number of settlements in the districts was obtained from the Federal State Statistics Service website (https://rosstat.gov.ru/). All shares were calculated based on the total area of the districts. The explanatory variables included in the analysis are presented in Table 1.

All variables were initially analyzed for mutual correlation using the nonparametric Spearman correlation test with a threshold value of *r*_s_ = 0.7 to avoid multicollinearity. In each pair of correlated variables, the one demonstrating the lowest correlation with other variables was retained for analysis [23].

### 2.4. Regression Analysis

In our study, we tested two regression approaches to examine the possible relationship between the cumulative intensity of ASF outbreaks in the model districts and various explanatory factors, including wild boar population density. In both cases, the response variable was the total annual number of ASF outbreaks in wild boar in the district [24].

One of the tested regression models was the negative binomial regression (NBR) model (NBRM), which is traditionally used to analyze count data with overdispersion [25, 26]. Additionally, this regression was used to investigate the significance of only one factor, namely the density of the wild boar population itself, in each model region.

As an alternative, we used the classification and regression tree (CART) model [27]. This approach is more flexible regarding variables of different scales and the potential presence of nonlinear relationships between the explanatory variables and the response variable [23].

Regression modeling was conducted simultaneously for two territories: the European part of Russia and the Far Eastern region.

#### 2.4.1. NBRM

The NBRM is a specific type of regression used for count data when the variation of the response variable exceeds its mean (i.e., when overdispersion is observed) [28, 29]. The choice of NBR in our case was justified by the distribution of the number of outbreaks in wild boars across municipal districts, where the mean is 1.84 and the variance is 38.41.

To ensure a stable and reliable selection of variables for the regression model, we applied the Lasso method. Lasso regression is a type of linear regression that introduces a regularization penalty to the loss function during training. This penalty is proportional to the absolute value of the coefficients, encouraging the model not only to fit the data but also to minimize the magnitude of the model weights. This characteristic makes Lasso regression particularly effective for feature selection, as it can reduce the number of features by setting the coefficients of less important variables to zero. All variables with zero coefficients were excluded from further analysis. The analysis was conducted in the R programing environment. The significance of the variables was assessed using Student's *t*-test with the corresponding *p*-value (a *p*-value ≤ 0.05 indicates sufficient statistical significance of the variable as a predictor in the regression model). The overall model fit quality was evaluated using the coefficient of determination *R*^2^, which represents the proportion of variance of the response variable accounted for by the model.

The spatial distributions of both model residuals were assessed using Moran's *I* spatial autocorrelation test, which demonstrates the correspondence between the observed spatial distribution of the analyzed variable and a hypothetical random distribution. Moran's *I* value close to zero, corresponding to low *z*-scores (*p*  > 0.05), indicate a near-normal distribution. The presence of spatial autocorrelation in the residuals indicate an unexplained clustering of the phenomenon under study that is not accounted for by the explanatory factors [30].

The negative binomial model with all selected factors was applied to the European and Far Eastern model regions.

#### 2.4.2. CART Analysis

CART analysis is a nonlinear nonparametric model constructed by binary partitioning a multidimensional set of covariates [26, 31].

At each step, the CART model divides the observations using a simple decision rule (e.g., if the wild boar density is less than 0.025 individuals/km^2^, then other variables need to be considered; if the density is above 0.025 individuals/km^2^, the risk of an outbreak increases and the presence of other predictors has negligible weight). This rule was chosen to minimize the diversity (regarding the binary outcome or classification) in the right and left “child nodes.” Branches and nodes are added until a stopping criterion is reached, and the tree is completed with “leaves” or “bins,” which contain the proportions of correctly and incorrectly classified observations [32].

The Gini index was used as the variable splitting method, and 10-fold cross-validation was applied to assess the predictive power of the resulting trees. The CART automatically performs cross-validation by growing the maximum number of branches on subsets of the data and then calculating error rates based on the unused portions of the dataset.

The completed CART analysis resulted in a “tree” with multiple partitions or branches depicted as branches. The independent variables and their splitting points are chosen to optimize a given suitability criterion, such as minimizing the residual sum of squares (MSE) applied to continuous data [33].

Regression and classification trees have advantages over other types of regression analysis in that they can handle various types of explanatory variables and do not require any data transformations [34, 35]. Compared to approaches based on linear regression, an advantage of CART analysis is that it can account for nonlinear relationships between the dependent variable and the set of explanatory factors. Missing or unaccounted values of independent variables have little impact on the outcome of the analysis. Thus, the method utilizes the best available information in the absence of variable values. In datasets of acceptable quality, this allows for the inclusion of all observations. CART methods provide a visual representation of the decision tree, which is intuitive and likely to be more acceptable to those unfamiliar with statistical analysis. The tree diagrams generated from the CART analysis can help structure explanations of the predictions.

The CART analysis was conducted for the final variables by region of the subjects of the RF using the rpart package [36], implemented in the R programing environment.

#### 2.4.3. Comparison of Regression Methods: Indicators for Evaluating the Performance of Models

The quality assessment of the constructed predictive regression models, examining the dependance of ASF outbreak intensity among wild boars on a set of risk factors, was conducted using *k*-fold cross-validation. This method is based on partitioning the data into a training set, used for model training, and a validation set, used to evaluate the prediction error.

To evaluate the predictive capability of the models, statistical indicators such as *R*^2^ (coefficient of determination), root mean square error (RMSE), and mean absolute error (MAE) were used [36–38].

1. The *R*-squared (*R*^2^) represents the square of the correlation between the observed outcome values and the predicted values of the model. The higher the adjusted *R*^2^, the better the model.
2. The RMSE measures the average forecasting error made by the model when predicting the outcome of observations. It reflects the average difference between the observed known outcome values and the values predicted by the model. The lower the RMSE, the better the model.
3. The MAE is an alternative to RMSE that is less sensitive to outliers. It corresponds to the average absolute difference between observed and predicted outcomes. The lower the MAE, the better the model.

In our study, we applied 10-fold cross-validation to evaluate the quality of the constructed models [39]. The performance quality assessment of the regression models was conducted using the “caret” package in the R software.

### 2.5. Software

Preliminary data processing and evaluation were conducted using Microsoft Office Excel (Microsoft Corporation, Redmond, Washington, USA). Visualization of the ASF epizootic situation and the wild boar population density was performed using ArcMap 10.8.2 (Esri, Redlands, California, USA). The statistically-oriented programing environment R (R Core Team, 2023) was used for regressionmodeling.

## 3. Results

### 3.1. Descriptive Analysis

**Figure 2:**
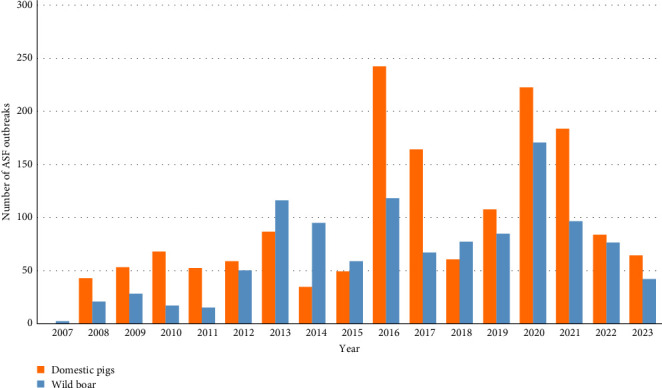
Yearly distribution of ASF outbreaks in wild boars and domestic pigs in the Russian Federation, 2007–2023.

During the analyzed period (from 2007 to 2023), a total of 1711 outbreaks of ASF among wild boars were registered in the model regions of Russia, including 1308 in the European part and 403 in the Far Eastern region. This accounted for 41.7% of all ASF outbreaks in the country during these years. The highest number of outbreaks among wild boars was recorded in the years 2013 (116 outbreaks), 2016 (118 outbreaks), 2020 (170 outbreaks), and 2021 (104 outbreaks) (Figure 2).

Geographically, massive outbreaks of ASF among wild boars were concentrated in the following subjects: Ryazan, Moscow, Tula, Tver, Vladimir, Smolensk, and Samara regions, as well as in the Pskov and Leningrad regions, which are adjacent to the border with Estonia. In the Far East, long-term persistence of the ASF virus has been noted in Primorsky Krai and in border regions—local areas where the population density of wild boars remains relatively high (Figure 1).

There are epidemiological features of the infection's manifestation in different regions. In some areas, the ASF outbreaks in wild boar were sporadic [38] (e.g., in Nizhny Novgorod Oblast) [19], while in other regions, it has been characterized by short-term but large-scale epizootics with widespread distribution across a significant part of the affected area. For instance, in Samara Oblast, such a massive epizootic occurred in 2020, with 60 ASF outbreaks in wild boars recorded over the year [39].

### 3.2. Regression Modeling Results

#### 3.2.1. NBRM

The NBRM applied to the regions of the European part of the RF (Zone 1 at Figure 1) identified the following significant factors associated with the intensity of ASF outbreaks among wild boars: the proportion of forest cover, the number of ASF outbreaks in domestic pigs, and the density of the wild boar population (the latter with a marginal significance level of *p*=0.05) (Table 2). The spatial autocorrelation test on the model residuals returns Moran's *I* coefficient of −0.265 (*p*=0.532) suggesting near-normal distribution of the residuals.

In the regions of the Far Eastern model zone (Zone II at Figure 1), the following significant factors were identified: the number of wild boars found dead, the number of ASF outbreaks among domestic pigs, and the density of the wild boar population (the latter with a marginal significance level of *p*=0.05) (Table 3). The spatial autocorrelation test on the model residuals returns Moran's *I* coefficient of −0.027 (*p*=0.432) suggesting near-normal distribution of the residuals.

The regression analysis with only a single factor (the density of the wild boar population) conducted for each of the model regions showed that for 17 out of 39 regions, the density of the wild boar population was a statistically significant predictor of the intensity of outbreak occurrences (Table 4).

#### 3.2.2. CART Analysis

**Figure 3:**
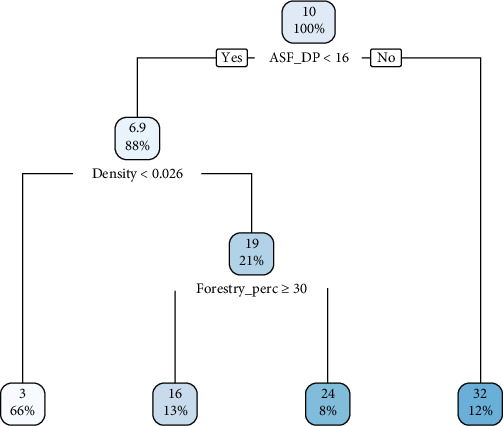
CART diagram of the regression model of ASF in wild boar in the regions of the European part of Russia.

**Figure 4:**
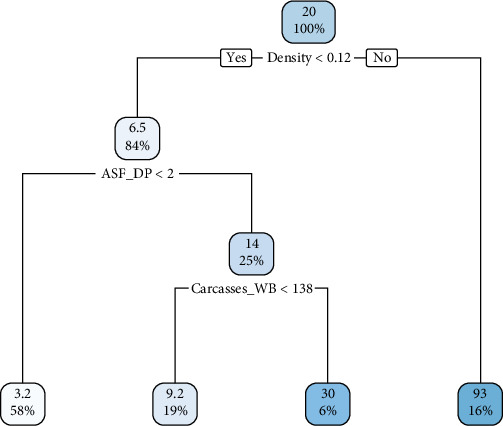
CART diagram of regression model of ASF in wild boar in the regions of the Russian Far East.

The classification tree diagrams constructed using CART for the study of ASF outbreaks in wild boar intensity in both the European and the Far Eastern parts of Russia are presented in Figures 3 and 4, respectively.

For the model pertaining to the European part of the RF, the significant factors were (in order of significant descend): the presence of outbreaks among domestic pigs, the density of the wild boar population, and the proportion of forest cover. The most significant node is represented by the occurrence of outbreaks among domestic pigs, suggesting that ASF in the wild population primarily arises in the presence of a certain number of outbreaks among domestic pigs. It should be noted that the significance of the threshold wild boar population density for this area is indicated by a cutoff point of 0.026 animals/km^2^. The third most significant factor in the model for the European part of Russia was the indicator of forest cover in the region, suggesting that with more than 30% forest coverage, there were 16 cases (13%) where the ASF outbreaks among wild boars persisted even with a low number of outbreaks among domestic pigs.

In the model for the Far Eastern part of the RF, the most significant factor is the density of the wild boar population, indicating that at densities above 0.12 individuals/km^2^, the highest proportion of outbreaks occurred without additional conditions. At densities lower than 0.12 individuals/km^2^, additional factors contributing to the occurrence of outbreaks in wild boars included the presence of ASF in the domestic pig population as well as a considerable number of found carcasses or remains of wild boars from which genetic material of the ASF virus was isolated.

When more than two ASF outbreaks are registered in the domestic pig population, the model, determined by the outcome variable of ASF outbreak intensity among wild boars, establishes a dependency of the epizootic on the number of found dead carcasses or remains of animals. This factor is represented by a final cutoff point of 138 individuals and was the concluding factor in determining significant elements of the epizootic identified through the modeling process.

Table 5 presents the predictive significance of risk factors for ASF among wild boars, determined by the significance weights (%), identified in the CART models for the two studied geographical territories.

#### 3.2.3. Comparative Metrics of Predictive Ability of Negative Binomial and CART Regression Models

The quality assessment indicators for the fit of the NBR and CART models, applied to reveal the risk factors for ASF outbreaks among wild boars in the European part and the Far Eastern region of the RF, are presented in Table 6, where, for each model, training and validation performance metrics are provided.

## 4. Discussion

Despite the efforts of scientists from many countries to develop a safe and effective vaccine against ASF, the current strategy for eradicating this disease relies on assessing the risks posed by identified factors that contribute to the spread of the infection, as well as strict adherence to biosecurity measures in animal husbandry. Most actions for the eradication and prevention of ASF are based on traditional principles of disease control, including epidemiological surveillance, investigation and destruction of infected herds, establishment of disease control zones, and movement restrictions and control of wild boar population, which may include fencing, depopulation, and passive and active monitoring measures.

Particular interest lies in analyzing the risk factors that facilitate the spread of the disease among wild boars, including the potential introduction of ASF into areas that are currently free of the virus [6]. The ecological cycle involving wild boars and the presence of the ASF virus in the environment is a major issue in contemporary ASF epidemiology, as not all mechanisms for the persistence of the pathogen in affected areas have yet been uncovered [7, 8].

Currently, there are extensive discussions regarding the significance of wild boar population density in the spread of ASF during localized introductions of the pathogen. Based on the experiences of European countries studying wild boar population density, this relationship is present but does not always hold primary importance in the occurrence of ASF outbreaks [5, 19, 20].

The key objective of this study was to utilize existing information on the population size and density of animals, as well as recorded ASF outbreaks among wild boars, available to professional epidemiologists, to identify the main risk factors contributing to the further expansion of the virus. A number of potential explanatory variables were tested using various analytical approaches, including linear NBR and CART analysis.

The comparative analysis of prediction errors from models employing different methodological approaches demonstrated a slight advantage for the CART model. This approach facilitates the development of clearer and more explainable regression structures for forecasting the significance of risk factors for ASF outbreaks among wild boars in affected regions of Russia.

Modeling results indicated that the occurrence of outbreaks among domestic pigs is the primary factor associated with ongoing local ASF epizootics in wild boars. The failure to confirm the reverse hypothesis—that wild boar cases influence outbreaks in domestic pigs, as previously tested [40]—may suggest that ASF in wild fauna plays a secondary role in the transmission dynamics. The factor of the number of wild boar remains found dead from various reasons emerged as significant in the models for the Far Eastern territory. On one hand, this relationship demonstrates a natural pattern in the frequent finding of remains in areas affected by ASF epizootics. On the other hand, it underscores the importance of enhanced passive monitoring measures based on the search for and removal of fallen boar carcasses. Indirectly, this may indicate that the remains of wild boars serve as a natural reservoir for the ASF virus, posing a threat in terms of maintaining the circulation of the virus. It should also be noted that the identified relationships are influenced by the quality of the raw data on reported cases in both domestic and wild pigs, which may be subject to underestimation.

The factor of forest cover proportion, which demonstrated significance in the model for the European territory of the RF, indicates a natural tendency for the development of ASF epizootics in areas with a larger habitat areas suitable for wild boars. In contrast, for the Far Eastern territory, this factor was not among the significant variables due to the generally denser forest cover in this region. Specifically, the range of forest cover proportion for the European territory is 33% ± 29%, whereas for the Far Eastern territory, it is 64% ± 27%.

This fact can also explain the higher threshold density value for wild boar as a risk factor in the Far Eastern territory. The identified threshold value for the European territory (0.026 animals/km^2^) almost exactly coincides with the density threshold recommended by the ASF control strategy in Russia (0.025 individuals/km^2^).

Based on the modeling results for individual subjects of the RF experiencing prolonged ASF outbreaks among wild boars, the subjects listed in Table 3 were identified, where a significant relationship was established between ASF outbreaks among wild boars and population density over time. Despite this, ASF outbreaks in wild boars were recorded in several subjects even where the population density was significantly lower than the recommended value of 0.025 individuals per km^2^, as stipulated by the order of the government of the RF dated September 30, 2016, Number 2048-r (with amendments from February 4, 2021) “On the Action Plan for Preventing the Introduction and Spread of ASF in the Territory of the RF.” This indicates that while wild boar population density plays an important role in the spread of the infection, it cannot be considered a primary factor, and control of ASF in this population should not be limited to depopulation measures only [13].

It should also be noted that the population density of wild boars is a value characterized by a high degree of uncertainty. Several reasons for this uncertainty may include: (1) the unreliability of primary data on wild boar population counts due to the inadequate reliability of the accounting methods used [13]; (2) the use of the total area of a region as a denominator in calculations can lead to inaccuracies, as the actual distribution area of wild boars may be much more compact, resulting in locations with localized high densities. Seasonal migrations of wild boars also play a significant role, leading to constant fluctuations in areas of increased local animal density.

The identified relationship somewhat accounts for the patterns of the epizootic process that determine the role of the susceptible population in the transmission of infection. The higher the level of the existing density of the susceptible population in the infection focus, the faster and more extensive the spread of the infection occurs, making it more challenging to control the process.

In cases of reduced wild boar populations, there is a possibility of infectious material persisting in the environment, creating conditions for a sustained epizootic process of ASF in specific, geographically constrained areas, leading to the formation of endemic regions. These areas are also difficult to combat and control with the standard preventive measures typically employed for ASF eradication [4, 5, 41].

## 5. Conclusion

A comparative analysis of the results from the constructed regression models leads to the following key conclusions:

1. The presence and number of ASF outbreaks among domestic pigs are determinants of the presence of ASF among wild boars in the study area.
2. The density of wild boar populations is a significant, albeit not decisive, factor in the development of epizootics in wild boar.
3. The regression approach using the CART method is a reliable modeling tool that provides more easily interpretable results.
4. To enhance the reliability of predictive models, efforts are needed to develop and implement more advanced methods for estimating wild boar population numbers.

## Figures and Tables

**Table 1:** Explanatory variables influencing the spread of African swine fever based on literature data.

Variables	Measurement units	Importance of the factor in the ASF epidemic	References
Share of forest cover	%	The presence of large forest areas is associated with wild boar habitats, which increases the likelihood of detecting sick animals.	[9, 10, 22, 23]

Share of water bodies	%	The presence and proximity to water bodies are associated with wild boar watering locations.	[9, 23]

Cropland areas with shrub vegetation	km^2^	The height of the meadow and shrub vegetation exceeding 1.5 m is associated with the identification of infected animals.	[23]

Altitude	m	High probability of detecting infected animals in optimal habitat.	[4, 5]

Population density	Person/km^2^	High population density is associated with infection of ASF.	[9]

Density of settlements, including villages	Unit/km^2^	Presence of villages increases the frequency of hunters' visits, which in turn may influence the detection of sick animals; increases the chance of ASF presence in small farms and backyards.	[4, 5, 9, 22, 23]

Number of wild boars found dead from various reasons	Head	The probability of detecting infected boar increased with the increase of monitoring activity.	[4, 5]

Length and density of the road network	km and km/km^2^	A dense road network increases the accessibility of an area by hunters and can increase the detection of infected animals, and is also an indirect indicator of economic activity of the population.	[4, 5, 9, 23]

Number of small-scale pig farms	Unit	An increase in the number of pig farms, especially small ones, is associated with an increased frequency of infected domestic animals.	[4, 5]

ASF outbreaks in domestic pigs	Unit	Proximity to outbreaks in domestic increases the likelihood of between-population contacts.	[4, 5]

Wild boar population density	Animals/km^2^	High boar density is directly related to the likelihood of disease occurrence.	[4, 5, 9, 23]

**Table 2:** Results of the negative binomial regression analysis of ASF outbreaks in wild boar with regard to risk factors in the European part of the Russian Federation (2007–2023).

Variables	Estimate	95% CI (confidence interval)	Standard error	*p*-Value
Forest cover percentage (%)	0.657	0.356–0.896	0.342	<0.001
Number of ASF outbreaks in domestic pigs	1.032	0.463–1.936	0.657	0.003
Wild boar population density (animals/km)^2^	1.006	0.387–1.894	0.415	0.050

**Table 3:** Results of the negative binomial regression analysis of ASF outbreaks in wild boar with regard to risk factors in the Far East of the Russian Federation (2007–2023).

Variables	Estimate	95% CI (confidence interval)	Standard error	*p*-Value
Number of wild boars found dead (animals)	0.354	0.278–0.932	0.234	0.001
Number of ASF outbreaks in domestic pigs	1.682	0.338–2.328	0.853	<0.001
Wild boar population density (animals/km)^2^	1.206	0.437–1.745	0.632	0.025

**Table 4:** Dependance of the number of ASF outbreaks in wild boar on animal population density in the regions of the Russian Federation, 2007–2023 (only the regions with *p* ≤ 0.05 are listed).

Subject	Estimate	95% CI (confidence interval)	Standard error	*p*-Value
Kaluga Oblast	5.565	1.421–15.634	1.811	0.002
Novgorod Oblast	8.834	0.679–19.679	3.869	0.022
Orenburg Oblast	34.163	5.325–25.456	15.493	0.027
Oryol Oblast	13.296	3.911–28.044	5.807	0.022
Republic of Chechnya	4.345	1.658–2.213	3.211	0.032
Republic of Chuvashia	39.070	7.579–91.482	12.047	0.001
Rostov Oblast	33.495	31.944–135.024	0.785	<0.001
Samara Oblast	27.656	10.487–50.677	8.844	0.001
Saratov Oblast	7.278	2.269–15.414	2.167	<0.001
Stavropol Krai	87.722	64.026–110.587	11.827	<0.001
Ulyanovsk Oblast	96.345	12.109–260.338	35.817	<0.001
Vladimir Oblast	13.059	1.244–25.423	3.137	<0.001
Volgograd Oblast	18.234	5.341–21.231	5.342	<0.001
Tver Oblast	8.274	3.661–14.281	1.539	<0.001
Amur Oblast	21.052	9.438–34.887	9.438	<0.001
Primorsky Krai	1.051	0.123–4.063	0.713	0.014
Khabarovsky Krai	6.870	0.01–10.456	3.398	0.043

**Table 5:** Significance weights of explanatory variables for the CART regression model in the European part and the Far Eastern regions of the Russian Federation.

Variable	CART model for the European part of Russia	CART model for the Far East part of Russia
Number of ASF outbreaks in domestic pigs	88%	83%
Wild boar population density (individuals/km)^2^	78%	87%
Forestry cover percentage (%)	56%	Not significant
Number of wild boars found that died from ASF (individuals)	Not significant	75%

**Table 6:** Performance characteristics of the CART and NBRM models.

Goodness of fit metrics	CART model				NBR model			
	Training I	Validation I	Training II	Validation II	Training I	Validation I	Training II	Validation II
Root mean square error (RMSE)	11.32	9.52	10.54	9.34	14.05	12.32	10.81	9.215
Mean absolute error (MAE)	2.55	2.231	3.634	3.427	2.567	2.474	3.845	3.324
Determination coefficient (*R*^2^)	0.61	0.65	0.58	0.71	0.59	0.61	0.63	0.64

*Note:* CART I and NBR I, regression models of European part of the Russian Federation and CART II and GLM II, regression model of Far Eastern part of the Russian Federation.

## Data Availability

The data on ASF cases in wild boar in Russia are publically available from international online databases (e.g., WOAH WAHIS, and FAO Empres-i). The data on the wild boar population in Russia can be obtained from the corresponding author upon a reasonable request.
